# Effect of SARS-CoV-2 vaccination on the outcomes of assisted reproductive technology: A review

**DOI:** 10.1097/MD.0000000000039310

**Published:** 2024-08-16

**Authors:** Xiaoli Kong, Chaoyan Shen, Tao Liu, Aijun Yang, Xu Liu, Fangyu Hou, Wei Wang, Sanhui Yang, Zewu Li, Jingwen Wang

**Affiliations:** a Department of Clinical Medicine, Jining Medical University, Jining, Shandong, China; b Jining No.1 People’s Hospital, Jining, China; c Affiliated Hospital of Jining Medical University, Jining, China.

**Keywords:** artificial insemination, assisted reproductive technology, COVID-19, in vitro fertilization and embryo transfer, intra cytoplasmic sperm injection, preimplantation genetic testing, SARS-CoV-2 vaccine

## Abstract

It has been over 4 years since the coronavirus disease 2019 outbreak, caused by severe acute respiratory syndrome coronavirus 2 (SARS-CoV-2). As an effective response to coronavirus disease 2019, the SARS-CoV-2 vaccines have been widely used around the world. However, couples who are planning to conceive naturally or by assisted reproductive technology (ART) are concerned about the impact of SARS-CoV-2 vaccines on pregnancy and offspring safety. Furthermore, in the initial stage of the epidemic, opinions among physicians and healthcare providers on whether ART patients should be immunized are divided due to the lack of data regarding the impact of the SARS-CoV-2 vaccine on ART. This is not the first, nor will it be the last time humans confront pandemics. It is time to summarize the experience about the effect of the SARS-CoV-2 vaccine on the outcomes of ART, which can provide a reference for the future. This paper reviewed relevant research, and significant adverse effects of the SARS-CoV-2 vaccine on the outcome of ART have not been observed. Considering the increased risk of serious complications in pregnant women infected with SARS-CoV-2, timely vaccination may be a wiser choice.

## 1. Introduction

As of CEST on April 12, 2023, the severe acute respiratory syndrome coronavirus 2 (SARS-CoV-2) has caused more than 762 million confirmed cases of coronavirus disease 2019 (COVID-19), including around 6.89 million deaths globally. And there were 354,950 cases and 1545 deaths occurred in 1 week until CEST on April 17, 2023.^[1]^ According to the data from the China CDC, the number of SARS-CoV-2 infections in China on April 13, 2023, was 2036.^[2]^ Vaccination is one of the most effective measures to prevent serious complications and deaths caused by SARS-CoV-2. Ten types of vaccines against COVID-19 have met the World Health Organization (WHO) criteria for safety and efficacy.^[3]^ However, concerns about the side effects of vaccines have prevented people from getting immunized timely. Several studies indicated that the COVID-19 vaccination has been associated with the occurrence of some side effects such as seizure-like symptoms, some muscle symptoms and Bell Palsy in a limited number of specific populations.^[4,5]^ While researchers noted that these possible side effects are much less than the risks associated with the SARS-CoV-2 infection, and that some side effects were even higher in the infected group compared to the vaccinated group, there are still many people who are hesitant to vaccinate, which also includes couples who are preparing for pregnancy. Given the high risk of SARS-CoV-2 infection developing into severe disease in pregnant women, WHO recommends that pregnant women and those who are considering getting pregnant be vaccinated against COVID-19 as soon as possible.^[6]^ Although several studies have shown that no significant adverse effects of the SARS-CoV-2 vaccines on fertility have been observed,^[7–9]^ there is still hesitation about vaccination among people who are planning to conceive naturally and patients undergoing assisted reproductive treatment. The internet search volume about the SARS-CoV-2 vaccine and fertility has increased by 207.56% to 2943.7%,^[10]^ demonstrating people’s concern about the relationship between the SARS-CoV-2 vaccine and reproductive health. Wang et al^[11]^ found that 32.32 percent of 987 infertile couples surveyed delayed SARS-CoV-2 vaccinations. Hesitancy in vaccination was positively associated with concerns about the impacts on pregnancy.^[11]^ To provide a reference for clinical practice and couples who are undergoing assisted reproductive technology (ART), this paper reviewed relevant studies and summarized the influence of the SARS-CoV-2 vaccination on the outcomes of common assisted reproductive technologies.

## 2. Effect of SARS-CoV-2 vaccination on artificial insemination

### 2.1. Vaccination and artificial insemination

Artificial insemination is the deliberate introduction of sperm into a female’s cervix or uterine cavity to achieve a pregnancy through in vivo fertilization by means other than sexual intercourse or in vitro fertilization. At present, intrauterine insemination (IUI) is the most widely used type of artificial insemination in clinical practice. Xu et al^[12]^ investigated the influence of inactivated SARS-CoV-2 vaccine on IUI, which included 1000 IUI cycles. The study demonstrated that the vaccinated and unvaccinated groups were comparable in terms of ongoing pregnancy rate and clinical pregnancy rate regardless of whether the IUI was performed by artificial insemination with husband’s semen or artificial insemination with donor’s semen. Analysis showed no negative effect of the inactivated SARS-CoV-2 vaccine on the outcomes of IUI. A multicenter cohort study^[13]^ investigated the association between 3 types of SARS-CoV-2 vaccines (inactivated vaccine, adenovirus vaccine, recombinant vaccine) used in China and the outcome of artificial insemination. No association was observed between women’s vaccination status and IUI pregnancy rates. Subgroup analysis according to vaccine type revealed that vaccine types were also not associated with pregnancy rates. In addition, the time interval between vaccination and IUI treatment was not linked to pregnancy rates. This study suggests that the 3 types of vaccines used in China have no obvious effect on IUI pregnancy rates. Overall, the number of studies concerning the SARS-CoV-2 vaccines and IUI treatment is limited. According to the available studies, there is no negative impact of the adenovirus vaccine, inactivated vaccine, or recombinant vaccine on IUI pregnancy rates. As both of these studies were conducted in China and the majority of participants were Chinese Han populations, there is a lack of evidence about the influence of the SARS-CoV-2 vaccine on artificial insemination in other ethnic populations. Moreover, only partial types of vaccines have been studied currently, limiting the generalizability of the findings. According to the statistics from WHO,^[14]^ the most-vaccinated SARS-CoV-2 vaccine type is the mRNA vaccine. However, there has been no available evidence about the impact of mRNA vaccines on the outcome of artificial insemination. Additionally, although available studies have shown no negative effect of the SARS-CoV-2 vaccine on IUI pregnancy rates, there is still a lack of evidence on IUI live birth rates and neonatal outcomes after SARS-CoV-2 vaccination. More studies with larger sample sizes and longer follow-ups are needed to provide evidence in the future.

### 2.2. Vaccination and human gametes

Given that the gametes of infertile couples are an important factor influencing IUI treatment, studies concerning the SARS-CoV-2 vaccine and gametes are also informative for IUI practice. Castiglione Morelli et al^[15]^ explored the relationship between the SARS-CoV-2 vaccine and follicular fluid metabolism in infertile women. Analysis indicated that there was no significant difference in anti-müllerian hormone and antral follicle count between the vaccinated and control groups. High oxidative stress and inflammation were not observed in the follicular fluid of vaccinated women. All considered, folliculogenesis and oocyte quality did not appear to be compromised by vaccination. However, the small sample size of the study, with a total of 6 female patients in the vaccination group (3 with the Pfizer-BioNTech mRNA vaccine, 1 with the Moderna mRNA vaccine, and 2 with the Oxford/AstraZeneca viral vector vaccine), did not allow for further analysis by vaccine type. Larger sample sizes are still needed to analyze different vaccine types in the future. Bentov et al^[16]^ revealed that follicular function was not affected in the BNT162b2 mRNA vaccine group and that anti-SARS-CoV-2 IgG antibodies were detected in the follicular fluid of the vaccinated group. The study suggests that the mRNA vaccine is safe for female follicles. Karavani et al^[17]^ also found no significant differences in the number of retrieved oocytes obtained by ultrasound-guided ovarian puncture and mature oocytes between the BNT162b2 mRNA-vaccinated and unvaccinated groups.

In terms of male gametes, a multicenter retrospective cohort study^[18]^ showed semen parameters did not change before and after vaccination, and the fertilization rate was also similar. No difference was observed, even when analyzed in subgroups according to vaccine type (mRNA vaccine or viral vector vaccine). According to the study, the SARS-CoV-2 vaccine does not affect sperm quality and fertilization capacity of men undergoing ART, which is safe for men’s reproductive health. Lifshitz et al^[19]^ analyzed the semen of 75 fertile men vaccinated with the BNT162b2 SARS-CoV-2 mRNA vaccine. The results showed no abnormal results in all but 2 samples (one with oligozoospermia and 1 with reduced sperm motility). This abnormal percentage is significantly lower than the percentage of oligospermia reported by WHO (5%)^[20]^ in fertile men, suggesting that semen parameters are within the normal range specified by WHO after vaccination. Studies have not found the pathogenic effects of the COVID-19 vaccine on male semen. Several other studies have also demonstrated that mRNA vaccines do not affect semen parameters.^[21–23]^ Regarding the inactivated SARS-CoV-2 vaccine, studies have reported no apparent adverse effects on male fertility and no significant changes in semen parameters before and after vaccination.^[24,25]^

Preliminary indications from the above studies are that the SARS-CoV-2 vaccine has no significant effect on women’s gametes. However, the types of vaccines studied have mainly focused on mRNA vaccines and viral vector vaccines. Studies on other types of vaccines, for example, inactivated vaccines, are still lacking. In the case of men’s gametes, researchers revealed that mRNA vaccines, viral vector vaccines and inactivated vaccines do not impact semen parameters. Although some studies have shown changes in semen parameters following vaccination, these fluctuations were within the normal range. Similar to women, no significant adverse effects of the SARS-CoV-2 vaccine on men’s gametes were observed.

In summary, the inactivated vaccine, the adenovirus vaccine, and the recombinant vaccine had no negative impact on pregnancy rates of artificial insemination. More evidence about the influence of other types of vaccines on artificial insemination is needed. In addition, there is still a shortage of data about live birth rates and neonatal outcomes with the SARS-CoV-2 vaccine on artificial insemination, as most of the studies just observed pregnancy rates. Finally, available studies indicated that COVID-19 vaccines do not adversely affect the gametes of both men and women, which is an important factor in artificial insemination treatment. All in all, the COVID-19 vaccine does not compromise the outcome of artificial insemination treatment.

## 3. Effect of SARS-CoV-2 vaccination on in vitro fertilization and embryo transfer or intra cytoplasmic sperm injection

Natural human reproduction begins with the fertilized egg created through the union of sperm and oocyte. The fertilized egg continues to divide and develop into the embryo, which is the original form of the fetus. In vitro fertilization and embryo transfer is a series of artificial fertilization procedures in which the oocyte obtained by ultrasound-guided ovarian puncture and the sperm fuse to form the fertilized egg, which develops into the embryo in vitro and is then transferred to the mother’s uterus for implantation and growth to achieve conception. Intra cytoplasmic sperm injection (ICSI) is an important derivative of in vitro fertilization and embryo transfer and is now widely used in clinical practice. ICSI involves the microscopic injection of a single sperm into the cytoplasm of a mature oocyte to help fertilize it.

### 3.1. Evidence concerning multiple vaccine types

Jacobs et al^[26]^ analyzed the impact of the SARS-CoV-2 vaccination on IVF outcomes. The vaccines used in the immunization group were the 1273 mRNA vaccine (Moderna), the BNT162b2 mRNA vaccine (Pfizer-BioNTech), and the viral vector vaccine Ad26.COV2.S (Janssen). There were no differences between the vaccinated and unvaccinated groups in terms of ovarian reserve, ovarian response, the number of oocytes retrieved, and the number of useable embryos. The rates of ongoing pregnancy and miscarriage did not significantly differ between the 2 groups either. Presently, the SARS-CoV-2 vaccines did not appear to have deleterious effects on IVF cycle stimulation characteristics, embryological variables, or clinical outcomes in IVF. Albeitawi et al^[27]^ also indicated that the SARS-CoV-2 vaccines (mRNA vaccine, viral vector vaccine, and inactivated virus vaccine) had no significant influence on IVF/ICSI implantation rates, fertilization rates, and clinical pregnancy rates. However, neither of the 2 studies analyzed further subgroups according to vaccine type. Requena et al^[28]^ compared the outcomes of treatment according to different types of vaccines, showing no significant differences between viral vector vaccines and mRNA vaccines in terms of ovarian response, the number of retrieved oocytes, and fertilization rates. Dong et al^[29]^ found that the type of vaccine (inactivated SARS-CoV-2 vaccines vs recombinant subunit vaccines) and the time interval from vaccination to IVF treatment did not compromise embryo quality or clinical pregnancy rates. In summary, mRNA vaccines, viral vector vaccines, recombinant subunit vaccines, and inactivated SARS-CoV-2 vaccines have not yet been reported to have detrimental effects on clinical pregnancy rates in IVF. Currently, there are a limited number of studies on the influence of vaccine types on IVF/ICSI. More studies with larger sample sizes are needed to compare the impact of different vaccine types on IVF/ICSI outcomes. Moreover, information about live birth rates and neonatal outcomes is lacking in all of the above studies. Further evidence remains to be accumulated regarding the long-term influence of the SARS-CoV-2 vaccines on IVF/ICSI.

### 3.2. Evidence concerning mRNA vaccines

In terms of mRNA vaccines, a study by Avraham et al^[30]^ showed there was no obvious effect of SARS-CoV-2 mRNA vaccines on oocyte retrieval and IVF pregnancy rates. Similarly, Odeh-Natour et al^[31]^ investigated that the Pfizer-BioNTech mRNA vaccine did not impair IVF/ICSI fertilization rate and pregnancy rate. Adler Lazarovits et al^[32]^ found that a booster mRNA vaccine (Pfizer-BioNtech) did not affect IVF clinical pregnancy rate. Additionally, Aizer et al^[33]^ performed an analysis of frozen embryo transfer and demonstrated that the mRNA vaccine had no significant adverse effects on the rate of implantation, ongoing pregnancy, and clinical pregnancy after frozen embryo transfer. Orvieto et al^[34]^ compared data from 36 infertile couples before and after vaccination. No statistical differences in embryonic parameters and ovarian stimulation characteristics such as the total dose of gonadotropin used, the peak estradiol and progesterone levels were observed in patients before and after mRNA vaccination, suggesting the safety of the SARS-CoV-2 mRNA vaccine on ovarian reserve and subsequent IVF treatment. To summarize the studies on the SARS-CoV-2 mRNA vaccine, it is observed that the mRNA vaccine does not affect the ovarian response, embryo development, IVF/ICSI fertilization, and pregnancy rate. Similarly, studies about mRNA vaccines have mostly stopped at early pregnancy outcomes. Studies about the potential influence of the SARS-CoV-2 mRNA vaccines on live birth rates and neonatal outcomes after IVF/ICSI are still lacking.

### 3.3. Evidence concerning inactivated SARS-CoV-2 vaccines

In terms of inactivated SARS-CoV-2 vaccines, Huang et al^[35]^ explored the effect of the inactivated vaccine on the outcomes of fresh embryo transfer. The results showed that there was no significant difference between the vaccinated and unvaccinated groups in terms of the number of oocytes retrieved, good-quality embryo rate, and clinical pregnancy rate. In addition, the patients were divided into 3 subgroups according to the time interval (≤1, 1–2, >2 months) between vaccination and IVF, and the 3 subgroups remained comparable. The study suggests that inactivated SARS-CoV-2 vaccination appears to be safe for IVF. Likewise, a study by Wu et al^[36]^ also showed no detrimental effect of the inactivated SARS-CoV-2 vaccine on ongoing pregnancy rate in fresh embryo transfer, and therefore the authors recommended that women receive the inactivated SARS-CoV-2 vaccine before IVF treatment. The team^[37]^ subsequently explored the effect of the inactivated SARS-CoV-2 vaccine on frozen embryo transfer and followed up on live birth rates and neonatal outcomes. The study, which included 2574 infertile couples, showed that the live birth rate (42.9% vs 43.9%, *P* = 0.688), ongoing pregnancy rate (52.2% vs 52.7%, *P* = 0.875), and clinical pregnancy rate (54.7% vs 54.2%, *P* = 0.868) in the vaccinated group were similar to those in the unvaccinated group. The newborns’ birth length (49.9 ± 1.7 vs 49.3 ± 2.6 cm, *P* = 0.141) and birth weight (3053.8 ± 372.5 vs 3039.2 ± 496.8 g, *P* = 0.347) were also similar in the 2 groups. The time interval between vaccination and embryo transfer (<3, 3–6, >6 months), upon the analysis, demonstrated no discernible influence on IVF treatment and neonatal outcome. The study suggests that the inactivated SARS-CoV-2 vaccine does not impair live birth rates and neonatal outcomes in frozen embryo transfers. This is one of the few studies that tracked live birth rates and neonatal outcomes, demonstrating the safety of the inactivated vaccine for IVF outcomes. The study of Huang et al^[38]^ also supported the conclusion that the inactivated SARS-CoV-2 vaccine did not affect the clinical pregnancy rate and live birth rate in frozen embryo transfers. Furthermore, a subgroup analysis based on the time interval between vaccination and embryo transfer found that the time interval (≤2 vs > 2 months) did not influence ART outcomes. In conclusion, this study indicates that the inactivated SARS-CoV-2 vaccine poses no detrimental effect on pregnancy outcomes in frozen embryo transfer. In addition, studies have shown that inactivated SARS-CoV-2 vaccination in men is not associated with negative outcomes in terms of embryo quality and IVF/ICSI clinical pregnancy rates.^[39,40]^ Regarding the time interval between vaccination and ART treatment, some studies have shown that the time interval does not have an impact on the outcome of ART treatment.^[29,35,37,38]^ However, 1 study^[41]^ analyzed 3052 women who underwent fresh embryo transfer. The vaccination groups were divided into 4 subgroups of ≤30, 31–60, 61–90, and ≥91 days, according to the time interval between the first dose of vaccine and IVF. The results showed that the subgroups with an interval of ≤30 days and 31 to 60 days had significantly lower ongoing pregnancy rates, (34.3%; adjusted odds ratio [aRR] = 0.61; 95% CI: 0.33–0.91) and (36.2%; aRR = 0.63; 95% CI: 0.42–0.85) respectively. In the subgroup of 61 to 90 days, there was a slight reduction in the ongoing pregnancy rate, but the difference was not statistically significant. No risk of reduced ongoing pregnancy rate was observed in the ≥91-day subgroup (56.3%; aRR = 0.96; 95% CI: 0.88–1.04). The conclusion was that the first dose of inactivated SARS-CoV-2 vaccine 60 days before IVF was associated with a reduced pregnancy rate. The authors recommend that fresh embryo transfer may need to be delayed until at least 61 days after vaccination. In this study, patients who received the first dose of vaccine before IVF were included, whereas previous studies^[29,35]^ included patients who had completed 2 doses of vaccination before treatment. Therefore, this study included patients who received a second dose of vaccine during IVF, which may explain why the results of this study are inconsistent with previous studies. Despite the large total sample size, the relatively small sample sizes in the ≤30-day group (35 cases) and the 31 to 60 day group (58 cases) may also have led to different findings from previous studies. As it relates to clinical practice, more studies are needed in the future to confirm this conclusion and thus provide the best clinical advice for patients. To summarize the studies on inactivated vaccines and IVF/ICSI, inactivated vaccines showed no significant effect on early pregnancy outcomes in IVF. Furthermore, 1 study^[37]^ demonstrated that inactivated vaccines did not affect live birth rates and neonatal outcomes in frozen embryo transfers, further confirming the safety of inactivated vaccines for ART. However, most studies only tracked early pregnancy outcomes, and more studies are needed to provide evidence for long-term maternal and infant safety.

## 4. Effect of SARS-CoV-2 vaccination on preimplantation genetic testing

Preimplantation genetic testing (PGT) is a genetic screening performed on embryos created via IVF before the transfer, helping patients select embryos that do not carry genetic defects and obtain healthy offspring. According to the contents of the test, PGT can be divided into PGT for monogenic (PGT-M), PGT for chromosome structure rearrangement (PGT-SR), and PGT for aneuploidy (PGT-A). The influence of the inactivated SARS-CoV-2 vaccine on embryo ploidy was assessed by Huang et al,^[42]^ who analyzed the data of 133 women undergoing PGT-A. The results showed similar euploidy rates in vaccinated and unvaccinated groups (23.2% ± 24.6% vs 22.6% ± 25.9%, *P* = 0.768). And clinical pregnancy rates remained comparable between the 2 groups (75.0% vs 60.0%, *P* = 0.289). The study indicated that the inactivated SARS-CoV-2 vaccines have no detrimental impact on embryo ploidy and clinical pregnancy rates in PGT-A. This provides evidence for the safety of the inactivated vaccine for PGT-A. But the study only observed early pregnancy outcomes, and further research concerning live birth rates and neonatal outcomes needs to be accumulated. A study by Brandão et al,^[43]^ in which all patients underwent PGT-A, showed that the SARS-CoV-2 mRNA vaccine didn’t present a negative effect on embryo fertilization and clinical pregnancy rates. Similarly, another study,^[44]^ which also included patients undergoing PGT-A, suggested that the mRNA vaccine had no detrimental effect on early pregnancy outcomes. In summary, the studies above indicated that neither inactivated SARS-CoV-2 vaccine nor mRNA vaccines detrimentally affect PGT-A clinical pregnancy rates. The available studies provide preliminary evidence of the SARS-CoV-2 vaccine safety for PGT-A. However, there is a lack of statistics about the influence of the SARS-CoV-2 vaccines on PGT-M and PGT-SR. As PGT-A, PGT-M, and PGT-SR mainly differ in their test contents, it may be presumed that the SARS-CoV-2 vaccines are also safe for PGT-M and PGT-SR. More research is needed to confirm this in the long run. In addition, the types of vaccines studied primarily focused on inactivated and mRNA vaccines. And the number of studies is limited. Studies comparing the effects of different vaccine types on PGT are still lacking. Finally, most of the studies observed early pregnancy outcomes. Further evidence for long-term maternal and neonatal safety still needs to be accumulated.

## 5. Discussion

In terms of artificial insemination, studies available indicated that the inactivated SARS-CoV-2 vaccine, adenovirus vector vaccine, and recombinant protein vaccine had no significant negative impact on pregnancy rates. However, studies on live birth rate and neonatal outcomes are still lacking, which deserves attention in future studies. In addition, the types of vaccines studied are limited, and further evidence on the safety of different types of vaccines for artificial insemination still needs to be accumulated. Finally, the gametes of both infertile couples are one of the most important factors affecting IUI treatment. It has been shown that the SARS-CoV-2 vaccine has no harmful effects on the gametes of either gender. In conclusion, SARS-CoV-2 vaccination does not interfere with the outcome of artificial insemination.

With regard to IVF/ICSI, there is evidence that the SARS-CoV-2 mRNA vaccines, viral vector vaccines, recombinant protein vaccines, and inactivated vaccines present no adverse effects on clinical pregnancy rates. Moreover, a study has shown that inactivated SARS-CoV-2 vaccines do not affect live birth rates and neonatal outcomes of frozen embryo transfers, further confirming the safety of inactivated vaccines. However, most studies have only followed up on early pregnancy outcomes. More studies are needed to provide evidence for long-term maternal and infant safety. It is particularly noteworthy that 1 study demonstrated that the 1st dose of inactivated SARS-CoV-2 vaccine 60 days before IVF was associated with a reduced pregnancy rate.^[41]^ If the findings are further confirmed, then fresh embryo transfer may need to be delayed until at least 61 days after vaccination. As it relates to clinical practice, more studies are needed in the future to confirm this finding and thus provide the best clinical advice to patients.

Similar to artificial insemination, there are few studies about the effect of the SARS-CoV-2 vaccine on PGT. According to available studies, the inactivated SARS-CoV-2 vaccine did not affect PGT-A embryo ploidy and clinical pregnancy rates. The SARS-CoV-2 mRNA vaccine also reported a neutral effect on PGT-A clinical pregnancy rates. Studies investigating the possible effects of other types of vaccines on PGT are still lacking. The above studies provide preliminary indications for the safety of SARS-CoV-2 in PGT-A. However, there is an absence of studies about PGT-M and PGT-SR. Based on the principles of PGT, it can be assumed that the SARS-CoV-2 vaccines are also safe for PGT-M and PGT-SR, which need to be confirmed in the future.

In summary, the SARS-CoV-2 vaccination does not impact the outcomes of ART (Table 1). The American Society for Reproductive Medicine recommended that those who are planning to conceive or who are already pregnant should be encouraged to immunize themselves with the SARS-CoV-2 vaccine and booster dose.^[45]^ The European Society of Human Reproduction and Embryology also recommended timely vaccination of men and women with assisted reproductive planning.^[46]^ A growing number of studies have confirmed the safety of the SARS-CoV-2 vaccines, while the SARS-CoV-2 infection is associated with an increased risk of maternal death and serious complications.^[47]^ All things considered, the benefits of vaccination outweigh the unknown risks of vaccines.

**Table 1:** Outcomes of assisted reproductive technology after the SARS-CoV-2 vaccination.

Study	Number	Vaccine type	ART type	Ongoing pregnancy (%)			Clinical pregnancy (%)		
				Vaccinated	Unvaccinated	*P* value	Vaccinated	Unvaccinated	*P* value
Xu et al^[12]^	653	Inactivated	AIH AID	20.9 20.9	28.1 28.8	.17 .19	12.5 11.0	11.3 10.3	.6 .73
Wang et al^[13]^	4185	Inactivated, adenovirus, recombinant	AIH	NA	NA	NA	1.008 (0.786–1.293)*	1*	NA
Jacobs et al^[26]^	280	mRNA, viral vector	IVF	NA	NA	NA	45.8	53.6	0.79 (0.48–1.29)*
Albeitawi et al^[27]^	151	mRNA, viral vector, inactivated	IVF, ICSI	NA	NA	NA	32.3	46.0	1.78 (0.88–3.60)*
Dong et al^[29]^	735	Inactivated, recombinant subunit	IVF	NA	NA	NA	53.03, 43.75, 50.00†	51.99	.91
Avraham et al^[30]^	200	mRNA	IVF, ICSI	NA	NA	NA	32.8	33.1	.96
Huang et al^[35]^	730	Inactivated	IVF, ICSI	NA	NA	NA	59.1	63.6	.507
Wu et al^[36]^	1583	Inactivated	IVF, ICSI	36.0	39.9	.27	44.4	47.4	.39
Cao et al ^[37]^	2574	Inactivated	IVF, ICSI	52, 2	52.7	.87	54.7	54.2	.86
Huang et al^[38]^	1210	inactivated	IVF, ICSI	NA	NA	NA	58.5	60.8	.59
Huang et al^[42]^	133	inactivated	PGT-A	NA	NA	NA	75.0	60.0	.28
Brandão et al^[43]^	4162‡	mRNA	PGT-A	NA	NA	NA	70.6	70.4	0.93 (0.69–1.26)§
Aharon et al^[44]^	1205	mRNA	PGT-A	47.5	53.6	.13	59.5	63.7	.27

AID = artificial insemination with donor’s semen, AIH = artificial insemination with husband’s semen, ART = assisted reproductive technology, ICSI = Intra cytoplasmic sperm injection, IVF = in vitro fertilization and embryo transfer, NA = not available, PGT = preimplantation genetic testing, PGT-A = PGT for aneuploidy.

* Odds ratio (95% confidence interval).

† Clinical pregnancy rate of the 3 subgroups. According to the vaccination status of both partners in infertile couples, the vaccinated group was divided into 3 subgroups: subgroup A, both partners in infertile couples had received vaccines; group B, infertile women were vaccinated, and the male partners were unvaccinated; group C, infertile men received vaccines but the female partners were unvaccinated.

‡ The number of embryo transfers.

§ Adjusted odds ratio (95% confidence interval).

Regarding research gaps, firstly, most studies available only observed early pregnancy outcomes such as fertilization rate and clinical pregnancy rate, while studies on live birth rate and neonatal outcomes are still lacking. Evidence on long-term maternal and infant outcomes needs to be accumulated further. Secondly, the types of vaccines studied have mostly focused on mRNA and inactivated vaccines, with less evidence for other types of vaccines. Thirdly, most of the current studies have focused on IVF/ICSI, with a small number of studies on IUI and PGT. Studies with larger sample sizes and longer follow-ups may provide additional evidence about the question above.

In the early stages of the epidemic, because of the lack of evidence about the influence of the SARS-CoV-2 vaccine on ART, no recommendations on whether infertility couples attempting to conceive through ART should receive the SARS-CoV-2 vaccine can be made.^[48]^ However, this is not the first time that mankind suffered from the pandemic, nor will it be the last. This review summarizes the relevant literature to provide experience for people. The studies indicated that no significant adverse effects of the SARS-CoV-2 vaccines on ART outcomes have been observed. Considering the increased risk of serious complications in pregnant women infected by the SARS-CoV-2, timely vaccination seems to be a wiser choice.

## Author contributions

**Conceptualization:** Xiaoli Kong, Chaoyan Shen, Tao Liu, Aijun Yang, Wei Wang, Zewu Li, Jingwen Wang.

**Data curation:** Xiaoli Kong, Chaoyan Shen, Tao Liu, Xu Liu, Fangyu Hou, Wei Wang, Jingwen Wang.

**Formal analysis:** Xiaoli Kong, Xu Liu, Fangyu Hou.

**Investigation:** Xiaoli Kong, Chaoyan Shen, Tao Liu, Xu Liu, Fangyu Hou.

**Project administration:** Xiaoli Kong.

**Software:** Xiaoli Kong.

**Visualization:** Xiaoli Kong, Sanhui Yang.

**Writing—original draft:** Xiaoli Kong, Zewu Li.

**Writing—review & editing:** Xiaoli Kong, Chaoyan Shen, Tao Liu, Aijun Yang, Wei Wang, Sanhui Yang, Jingwen Wang.

**Supervision:** Chaoyan Shen, Tao Liu, Wei Wang, Jingwen Wang.

**Validation:** Chaoyan Shen, Tao Liu, Aijun Yang, Wei Wang, Sanhui Yang, Zewu Li, Jingwen Wang.

**Funding acquisition:** Wei Wang, Chaoyan Shen, Tao Liu, Jingwen Wang.
